# Long-Term Survival of a Patient With Peritoneal Carcinomatosis From Pancreatic Cancer Maintained by Nanoliposomal Irinotecan as Third-Line Chemotherapy

**DOI:** 10.7759/cureus.22355

**Published:** 2022-02-18

**Authors:** Keiji Nagata, Masatoshi Kajiwara, Takahisa Fujikawa

**Affiliations:** 1 Surgery, Kokura Memorial Hospital, Kitakyushu, JPN; 2 Gastroenterological Surgery, Fukuoka University Hospital, Fukuoka, JPN

**Keywords:** long-term survival, combination chemotherapy, nasoliposomal irinotecan, carcinomatosa peritoni, pancreatic cancer

## Abstract

Pancreatic cancer is still one of the most fatal neoplastic diseases, and the recurrence occurs in more than 80% of the patients even though radical resection is performed. We experienced a long-term survival case of a patient with peritoneal carcinomatosis from pancreatic cancer maintained by nanoliposomal irinotecan (nal-IRI) in combination with fluorouracil and folinic acid (FF) as third-line chemotherapy. Nal-IRI + FF combination chemotherapy is one of the promising options for the management of intractable recurrent pancreatic cancer.

## Introduction

Pancreatic cancer is still one of the most fatal diseases. Surgical resection is the only treatment that can be expected to cure pancreatic cancer, but the proportion of resectable cases is about 15%-20% [1,2]. Furthermore, there are many cases of recurrence even after radical resection. Chemotherapy is currently the main treatment for pancreatic cancer with distant metastasis, and nanoliposomal irinotecan (nal-IRI) is the first drug delivery system (DDS) preparation to be used for pancreatic cancer.

We report a case of peritoneal dissemination and abdominal wall metastasis from pancreatic cancer in which nal-IRI + fluorouracil and folinic acid (FF) combination therapy was effective as third-line chemotherapy and the disease could be controlled for a long period of time.

## Case presentation

**Figure 1:**
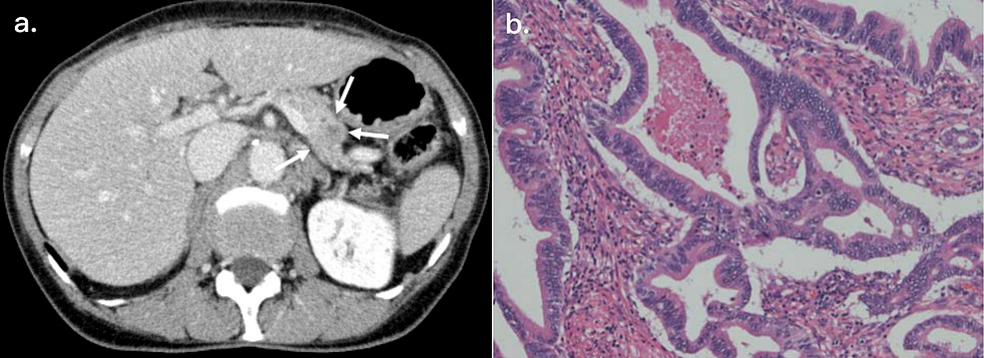
Tumor characteristics of the current case (a) Preoperative CT findings. Abdominal enhanced CT scan showed a hypovascular mass (arrows), approximately 22 mm in diameter, at the pancreatic tail, and an invasion to the splenic artery and vein was suspected. (b) Microscopic examination revealed moderately differentiated invasive ductal carcinoma of the pancreas accompanied by regional lymph node involvement. CT: Computed tomography.

A 70-year-old female presented with abdominal pain, and she was diagnosed with pancreatic tail cancer by computed tomography (CT) scan, magnetic resonance cholangiopancreatography (MRCP), and endoscopic retrograde cholangiopancreatography/endoscopic ultrasound (ERCP/EUS) (Figure 1, Panel a). She underwent radical distal pancreatectomy with regional lymph node dissection and revealed moderately differentiated invasive ductal carcinoma accompanied by regional lymph node involvement (Figure 1, Panel b).

**Figure 2:**
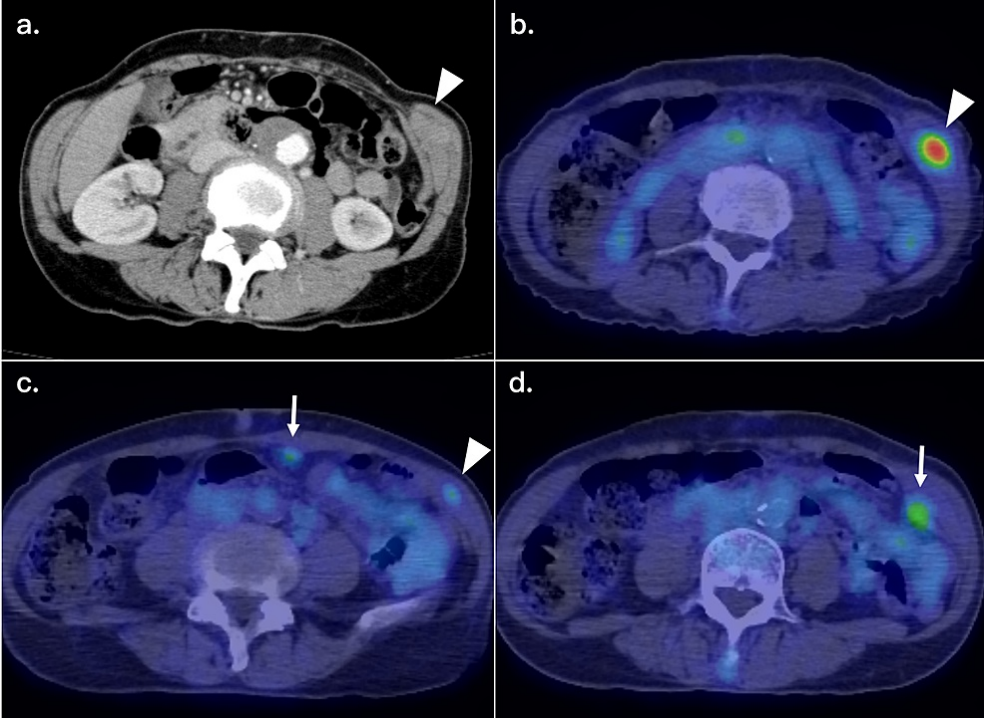
CT and PET-CT findings at the first recurrence (a, b) An enhanced CT scan showed a contrasted nodule in the left abdominal wall, with positive FDG uptake (SUVmax = 6.52). (c, d) PET-CT also showed multiple intraperitoneal and abdominal wall metastases with FDG uptake at the peritoneum (arrows) and in the abdominal wall (arrowheads). CT: Computed tomography; PET-CT: positron emission tomography-computed tomography; FDG: fluorodeoxyglucose; SUVmax: maximum standardized uptake value.

The postoperative course was uneventful, and the patient recovered without any complications. She subsequently received six-month adjuvant S-1 chemotherapy (six weeks/cycle, 80 mg/day of oral S-1 on days 1-28), and the stable disease-free status was maintained, although multiple intraperitoneal and abdominal wall masses were developed nine months after tumor resection (Figure 2).

**Figure 3:**
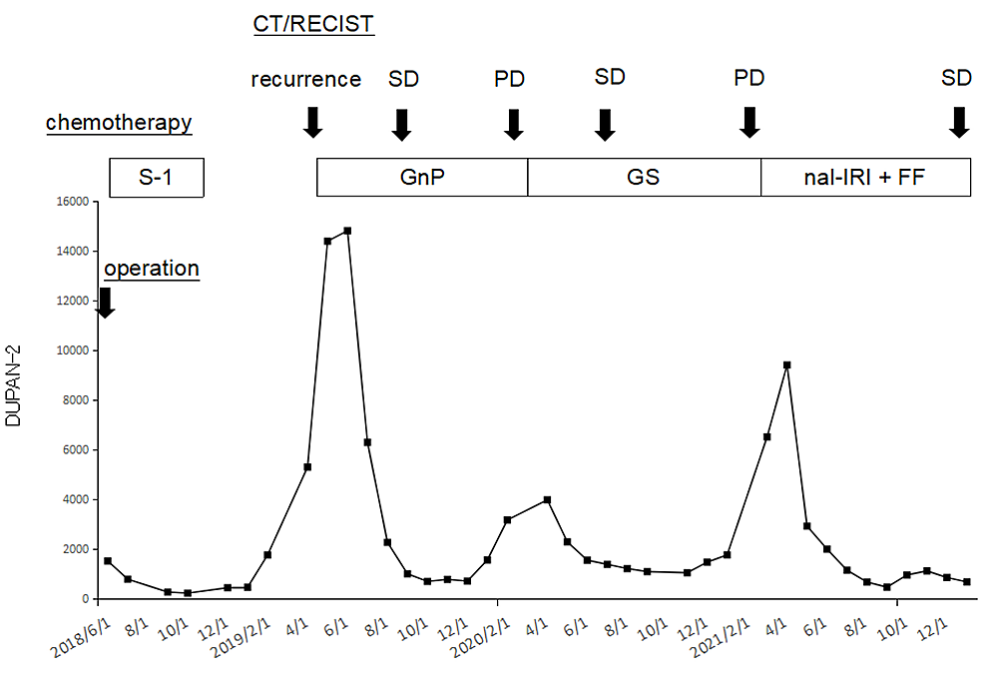
Clinical course of the current patient with pancreatic tail cancer The patient received six-month adjuvant S-1 chemotherapy after distal pancreatectomy. Multiple intraperitoneal and abdominal wall masses were developed nine months after tumor resection. First-line chemotherapy (GnP) was introduced, and her disease condition was SD for 12 months, but it later became PD. Second-line chemotherapy (GS) was introduced, and SD was maintained but became PD 11 months later. Third-line chemotherapy (nal-IRI + FF) was then initiated, and her tumor condition was maintained SD with a significant reduction of DUPAN-2 values for 12 months. GnP: Gemcitabine plus nab-paclitaxel; SD: stable disease; PD: progressive disease; GS: gemcitabine plus S-1; nal-IRI + FF: nanoliposomal irinotecan plus fluorouracil and folinic acid; RECIST: response evaluation criteria in solid tumors; DUPAN-2: duke pancreatic monoclonal antigen type-2; CT: computed tomography.

The whole clinical course of the current patient with serial changes in serum duke pancreatic monoclonal antigen type-2 (DUPAN-2) values was summarized in Figure 3.

**Figure 4:**
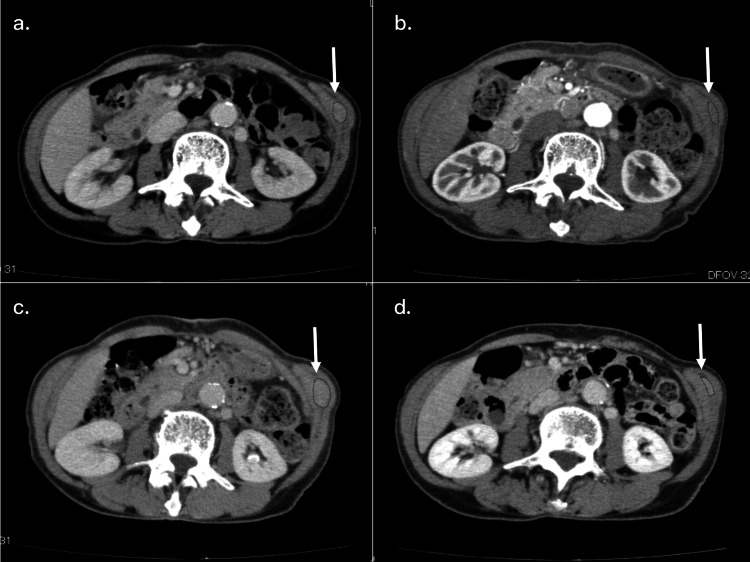
Sequential CT findings of the recurrent abdominal wall tumor (arrows and dotted areas) (a) The tumor in the left lateral abdominal wall was initially maintained as SD for 12 months after starting first-line chemotherapy but later became PD. The tumor was maintained as SD for 11 months after the introduction of second-line chemotherapy (b) but became PD thereafter (c). (d) Subsequently, third-line chemotherapy using nal-IRI + FF was introduced, and the tumor was maintained as SD for 12 months with an excellent physical condition. CT: Computed tomography; SD: stable disease; PD: progressive disease; nal-IRI + FF: nanoliposomal irinotecan plus fluorouracil and folinic acid.

First-line chemotherapy using gemcitabine plus nab-paclitaxel (GnP) (four weeks/cycle, 1000 mg/m^2^ [1200 mg] of gemcitabine plus 125 mg/m^2^ [150 mg] of nab-paclitaxel at days 1, 8, and 15) was introduced, and her disease condition was stable for 12 months but later became progressive (Figure 4, Panel a). Second-line chemotherapy using gemcitabine plus S-1 (GS) (three weeks/cycle, 80 mg/day of oral S-1 on days 1-14 plus 1000 mg/m^2^ [1200 mg] of gemcitabine at days 1 and 8) was introduced, and stable tumor condition was maintained (Figure 4, Panel b), but the tumors became progressive 11 months thereafter (Figure 4, Panel c).

Third-line chemotherapy was then scheduled using nal-IRI in combination with FF, and she was well tolerated without any severe adverse effects. Her height, body weight, and body mass index were 147.6 cm, 36.5 kg, and 16.8 kg/m^2^, respectively. She received 50 mg/m^2^ (60 mg) of nal-IRI plus 2400 mg/m^2^ (3000 mg) of fluorouracil and 200 mg/m^2^ (250 mg) of folinic acid every two weeks. The dose of nal-IRI was reduced to 50 mg/m^2^ because she was homozygous for UGT1A1*6 (uridine diphosphate glucuronosyltransferase 1A1 gene) by UGT1A1 genetic polymorphism analysis. Her tumor condition is currently maintained as a stable disease with a significant reduction of DUPAN-2 values, and it remains stable without tumor progression for 12 months (47 months after initial tumor resection) (Figure 4, Panel d).

## Discussion

Pancreatic cancer is still one of the most deadly cancers with a five-year survival rate of less than 20% even after curative resection [3,4]. Median survival for patients receiving surgical resection is only 12.6 months, and the high recurrence rate is the main reason for the very poor prognosis [5]. Recurrence and/or metastasis from pancreatic cancer is considered to present as multiple recurrent lesions or peritoneal dissemination [6,7], although some selected patients may have a chance of long-term survival, thanks to recent advances in combination chemotherapy [8,9].

As second-line chemotherapy after FOLFIRINOX (a combination of fluorouracil, leucovorin, irinotecan, and oxaliplatin) therapy or GnP therapy, nal-IRI was approved and launched for pancreatic cancer with distant metastasis. Irinotecan is hydrolyzed to the active metabolite SN-38 by carboxylesterase in vivo and inhibits DNA synthesis through inhibition of type I topoisomerase [10]. Nal-IRI is a nanoliposomal preparation of irinotecan modified with polyethylene glycol and has been reported to have the following effects: (1) increased tumor accumulation due to increased vascular permeability and retention [11] and (2) enhanced antitumor activity by prolonging the duration of SN-38 exposure within the tumor [12].

Nal-IRI + FF therapy showed a significant improvement in overall survival (OS) in phase III clinical trial (NAPOLI-1 study) compared to the FF group (median survival for nal-IRI + FF group: 6.1 months versus for FF group: 4.2 months, p = 0.012) [13]. The nal-IRI + FF combination therapy is considered to be a promising regimen as a new second-line chemotherapy option for metastatic pancreatic cancer that has progressed after gemcitabine-related regimens. Assi et al. [14] reported a long-term survival case with nal-IRI as second-line chemotherapy. In his paper, the patient received 58 cycles of nal-IRI + FF totally, which she tolerated very well without any dose adjustments until imaging showed evidence of PD. Relative to the nal-IRI + FF initiation date, the OS was 40 months. However, to the best of our knowledge, this is a rare case report that shows nal-IRI + FF therapy has been successful as third-line chemotherapy for metastatic pancreatic cancer and long-term disease control has been obtained.

In the current case, GnP therapy and GS therapy were performed as first-line and second-line chemotherapy for peritoneal dissemination and abdominal wall recurrence of pancreatic cancer, respectively, but the tumor condition became PD. Subsequently, nal-IRI + FF therapy was started as third-line chemotherapy, resulting in a significant reduction of DUPAN-2 levels, reduced size of the intraperitoneal and abdominal wall lesions, and achievement of long-term disease control. The tumor status of SD is still maintained as 12 months after the start of nal-IRI + FF therapy (47 months after tumor resection).

Glassman et al. [15] demonstrated the safety and effectiveness of nal-IRI + FF therapy in advanced pancreatic cancer patients. In his study, dose reductions were most frequently due to fatigue and diarrhea but were not associated with worse outcomes. In the present case, the patient received 20 cycles of nal-IRI + FF therapy and tolerated the regimen without any dose reductions. Her adverse effects included nausea and fatigue, all of which were managed with medications. Since nal-IRI + FF is still effective, further follow-up is necessary to determine the efficacy and tolerability of nal-IRI in the longer term. Nevertheless, the predictors of the effects of nal-IRI therapy are still unclear, and analysis by accumulating future cases is necessary.

## Conclusions

We reported a long-term survival case of a patient with peritoneal carcinomatosis from pancreatic cancer. After the failure of first-line and second-line gemcitabine-based chemotherapy, the patient was maintained with a stable tumor condition by nal-IRI + FF therapy as third-line chemotherapy for an additional 12 months. So, nal-IRI + FF combination chemotherapy is one of the promising options for the management of intractable recurrent pancreatic cancer.
